# Supplementary material for: MetaRibo-Seq measures translation in microbiomes
Source: Nat Commun. 2020 Jun 29;11:3268. doi: 10.1038/s41467-020-17081-z (PMC7324362; doi:10.1038/s41467-020-17081-z)
Supplement: Supplementary file 10 — Supplementary Data 7 [file 41467_2020_17081_MOESM10_ESM.zip › File2/Confidence_High_Taxonomy/266607_out.krona.html]

Javascript must be enabled to view this page.

members
magnitude
magnitudeUnassigned
count
unassigned
taxon
rank

266607\_out

214

214
superkingdom
2

214
phylum
976

class
200643
214

171549
order
214

family
2005525
1

genus
375288
1

species
46503
1

SRS049995\_contig\_number\_22888

815
family
213

genus

SRS011239\_contig\_number\_27806SRS012849\_contig\_number\_30693SRS013098\_contig\_number\_29719SRS013215\_contig\_number\_2295SRS013638\_contig\_number\_8861SRS013800\_contig\_number\_17500SRS013940\_contig\_number\_22548SRS013951\_contig\_number\_contig-100\_2425.221927SRS014287\_contig\_number\_contig-100\_2668.108320SRS014613\_contig\_number\_12306SRS014683\_contig\_number\_17801SRS015065\_contig\_number\_37528SRS015431\_contig\_number\_83327SRS015663\_contig\_number\_contig-100\_2325.194118SRS015854\_contig\_number\_17698SRS016018\_contig\_number\_11578SRS016897\_contig\_number\_contig-100\_2202.23771SRS018313\_contig\_number\_8758SRS018427\_contig\_number\_31203SRS018575\_contig\_number\_5494SRS018817\_contig\_number\_20099SRS018936\_contig\_number\_contig-100\_1645.85349SRS019030\_contig\_number\_17365SRS019068\_contig\_number\_113577SRS019397\_contig\_number\_3261SRS020394\_contig\_number\_contig-100\_3243.97173SRS021219\_contig\_number\_contig-100\_644.33270SRS023715\_contig\_number\_27147SRS024132\_contig\_number\_20583SRS043701\_contig\_number\_contig-100\_1996.108678SRS049959\_contig\_number\_51261SRS051031\_contig\_number\_35088SRS053573\_contig\_number\_437SRS054352\_contig\_number\_contig-100\_1710.136336SRS057478\_contig\_number\_6028SRS063518\_contig\_number\_38510SRS064276\_contig\_number\_27916SRS065176\_contig\_number\_11300SRS076976\_contig\_number\_20264SRS077024\_contig\_number\_contig-100\_1577.91678SRS077502\_contig\_number\_18315SRS078176\_contig\_number\_19867SRS078419\_contig\_number\_35123SRS078665\_contig\_number\_9073SRS097958\_contig\_number\_22818SRS098717\_contig\_number\_20867SRS101376\_contig\_number\_25375SRS1041031\_contig\_number\_30880SRS1041134\_contig\_number\_1272SRS1041136\_contig\_number\_17840SRS1041137\_contig\_number\_32083SRS1041138\_contig\_number\_contig-100\_2070.73365SRS104400\_contig\_number\_contig-100\_4513.304198SRS1055056\_contig\_number\_contig-100\_2511.74709SRS142503\_contig\_number\_contig-100\_3731.294802SRS142923\_contig\_number\_contig-100\_3419.115791SRS143780\_contig\_number\_18456SRS144537\_contig\_number\_contig-100\_3367.253689SRS146812\_contig\_number\_contig-100\_6581.260195SRS147977\_contig\_number\_contig-100\_757.40813SRS148091\_contig\_number\_contig-100\_583.90370SRS149181\_contig\_number\_22648SRS149244\_contig\_number\_7874SRS149784\_contig\_number\_contig-100\_1427.168029SRS893279\_contig\_number\_contig-100\_4312.86443SRS893288\_contig\_number\_4237SRS893295\_contig\_number\_contig-100\_2231.70390SRS893378\_contig\_number\_20348
816
213
68

338188
species
5

SRS063040\_contig\_number\_12844SRS063370\_contig\_number\_385SRS147088\_contig\_number\_3716SRS893292\_contig\_number\_4018SRS893300\_contig\_number\_4092


SRS014412\_contig\_number\_17239SRS015578\_contig\_number\_34163SRS017433\_contig\_number\_9387SRS017821\_contig\_number\_contig-100\_239.75762SRS019445\_contig\_number\_contig-100\_3173.174360SRS022524\_contig\_number\_14880SRS022713\_contig\_number\_740SRS050752\_contig\_number\_26869SRS056519\_contig\_number\_209SRS062427\_contig\_number\_1962SRS063190\_contig\_number\_13411SRS074964\_contig\_number\_contig-100\_2119.56521SRS077086\_contig\_number\_3660SRS1041038\_contig\_number\_15507SRS1041147\_contig\_number\_3862SRS144297\_contig\_number\_12569SRS145308\_contig\_number\_2232SRS147346\_contig\_number\_33433SRS893369\_contig\_number\_11780SRS893373\_contig\_number\_contig-100\_2102.152779SRS893382\_contig\_number\_5783
species
1262744
21

species
817
2

SRS014736\_contig\_number\_9093SRS050299\_contig\_number\_3200

7
species
1739517

SRS020508\_contig\_number\_364SRS046810\_contig\_number\_contig-100\_30.31SRS052576\_contig\_number\_259SRS1041141\_contig\_number\_14865SRS104485\_contig\_number\_216SRS147022\_contig\_number\_648SRS893259\_contig\_number\_4993

1
821
species

SRS019601\_contig\_number\_8718

1
species
1946086

SRS1041039\_contig\_number\_29180


SRS065397\_contig\_number\_8958SRS1055038\_contig\_number\_1494
291645
species
2

1
820
species

SRS054956\_contig\_number\_9894


SRS015190\_contig\_number\_3229SRS043411\_contig\_number\_6849SRS1041133\_contig\_number\_19295
469590
species
3


SRS075773\_contig\_number\_13540SRS076929\_contig\_number\_1597
species
246787
2


SRS062701\_contig\_number\_417SRS063928\_contig\_number\_536SRS1041142\_contig\_number\_5665SRS1055034\_contig\_number\_9104
457390
species
4

2
species
1262750

SRS046717\_contig\_number\_11126SRS098827\_contig\_number\_9117


SRS011405\_contig\_number\_262SRS016132\_contig\_number\_3867SRS016132\_contig\_number\_4571SRS018623\_contig\_number\_858SRS019178\_contig\_number\_5215SRS019381\_contig\_number\_14903SRS050925\_contig\_number\_16446SRS064973\_contig\_number\_contig-100\_620.17255SRS1041112\_contig\_number\_2385SRS105153\_contig\_number\_15262SRS140645\_contig\_number\_865SRS140645\_contig\_number\_2299SRS147272\_contig\_number\_649SRS147425\_contig\_number\_1674SRS893327\_contig\_number\_6173
15
371601
species

1739298
species
2

SRS103987\_contig\_number\_804SRS148159\_contig\_number\_42748


SRS016203\_contig\_number\_11246SRS022137\_contig\_number\_11374SRS045244\_contig\_number\_628SRS045826\_contig\_number\_2186SRS046068\_contig\_number\_3612SRS049773\_contig\_number\_10143SRS052697\_contig\_number\_24430SRS147614\_contig\_number\_4109
8
species
47678


SRS148511\_contig\_number\_11966
357276
species
1

2
1796613
species

SRS143895\_contig\_number\_34859SRS148196\_contig\_number\_contig-100\_576.255846

species
457387
1

SRS075984\_contig\_number\_20861

310300
species
2

SRS018984\_contig\_number\_321SRS019968\_contig\_number\_16172

47
28116
species

SRS012902\_contig\_number\_133SRS013476\_contig\_number\_40856SRS014979\_contig\_number\_35719SRS015369\_contig\_number\_3402SRS016381\_contig\_number\_1871SRS016840\_contig\_number\_2439SRS017191\_contig\_number\_6124SRS017622\_contig\_number\_47SRS017701\_contig\_number\_9853SRS019496\_contig\_number\_contig-100\_224.109314SRS019693\_contig\_number\_7531SRS023829\_contig\_number\_102SRS024549\_contig\_number\_663SRS046369\_contig\_number\_12581SRS050422\_contig\_number\_contig-100\_2904.184417SRS056537\_contig\_number\_69560SRS057480\_contig\_number\_8473SRS058268\_contig\_number\_16SRS063813\_contig\_number\_5932SRS064232\_contig\_number\_1503SRS075398\_contig\_number\_1180SRS077231\_contig\_number\_24810SRS098061\_contig\_number\_4377SRS098881\_contig\_number\_14414SRS104084\_contig\_number\_5287SRS1041092\_contig\_number\_9204SRS104693\_contig\_number\_12224SRS104975\_contig\_number\_14596SRS1055095\_contig\_number\_2218SRS142542\_contig\_number\_23513SRS142980\_contig\_number\_6446SRS143148\_contig\_number\_16666SRS143372\_contig\_number\_1485SRS143417\_contig\_number\_29425SRS143466\_contig\_number\_29569SRS143722\_contig\_number\_1881SRS144183\_contig\_number\_40906SRS145497\_contig\_number\_11469SRS147653\_contig\_number\_6420SRS148253\_contig\_number\_14031SRS148721\_contig\_number\_3903SRS148784\_contig\_number\_16522SRS148970\_contig\_number\_1636SRS149075\_contig\_number\_14549SRS893253\_contig\_number\_25SRS893256\_contig\_number\_1669SRS893358\_contig\_number\_1951

species
818
5

SRS012273\_contig\_number\_contig-100\_1610.266815SRS014459\_contig\_number\_19843SRS024075\_contig\_number\_10384SRS054905\_contig\_number\_17967SRS140513\_contig\_number\_3580


SRS055982\_contig\_number\_16276
1
species
291644

1
species
2292956

SRS050801\_contig\_number\_2645


SRS015960\_contig\_number\_8133
1
329854
species


SRS013382\_contig\_number\_1517SRS056519\_contig\_number\_327
2
species
2302926

1
species
2292957

SRS014855\_contig\_number\_5208


SRS022071\_contig\_number\_25108SRS050026\_contig\_number\_9735SRS053649\_contig\_number\_2324SRS056259\_contig\_number\_773
species
46506
4

species
1897051
1

SRS143085\_contig\_number\_3525
